# Evolution of the axial system in craniates: morphology and function of the perivertebral musculature

**DOI:** 10.1186/1742-9994-8-4

**Published:** 2011-02-10

**Authors:** Nadja Schilling

**Affiliations:** 1 Institute of Systematic Zoology and Evolutionary Biology, Friedrich-Schiller-University Jena, Germany

## Abstract

The axial musculoskeletal system represents the plesiomorphic locomotor engine of the vertebrate body, playing a central role in locomotion. In craniates, the evolution of the postcranial skeleton is characterized by two major transformations. First, the axial skeleton became increasingly functionally and morphologically regionalized. Second, the axial-based locomotion plesiomorphic for craniates became progressively appendage-based with the evolution of extremities in tetrapods. These changes, together with the transition to land, caused increased complexity in the planes in which axial movements occur and moments act on the body and were accompanied by profound changes in axial muscle function. To increase our understanding of the evolutionary transformations of the structure and function of the perivertebral musculature, this review integrates recent anatomical and physiological data (e.g., muscle fiber types, activation patterns) with gross-anatomical and kinematic findings for pivotal craniate taxa. This information is mapped onto a phylogenetic hypothesis to infer the putative character set of the last common ancestor of the respective taxa and to conjecture patterns of locomotor and muscular evolution. The increasing anatomical and functional complexity in the muscular arrangement during craniate evolution is associated with changes in fiber angulation and fiber-type distribution, i.e., increasing obliqueness in fiber orientation and segregation of fatigue-resistant fibers in deeper muscle regions. The loss of superficial fatigue-resistant fibers may be related to the profound gross anatomical reorganization of the axial musculature during the tetrapod evolution. The plesiomorphic function of the axial musculature -mobilization- is retained in all craniates. Along with the evolution of limbs and the subsequent transition to land, axial muscles additionally function to globally stabilize the trunk against inertial and extrinsic limb muscle forces as well as gravitational forces. Associated with the evolution of sagittal mobility and a parasagittal limb posture, axial muscles in mammals also stabilize the trunk against sagittal components of extrinsic limb muscle action as well as the inertia of the body's center of mass. Thus, the axial system is central to the static and dynamic control of the body posture in all craniates and, in gnathostomes, additionally provides the foundation for the mechanical work of the appendicular system.

## Introduction

The axial musculoskeletal system represents the plesiomorphic propulsive engine of the vertebrate body and maintains a central role in locomotion in all craniates. Considering its evolutionary antecedence to the appendicular system and its importance for locomotion, our understanding of the axial system is surprisingly limited compared to our understanding of the limbs.

The evolution of the axial system is marked by profound changes in its morphology and function. The increasing differentiation of its muscular, neural, and skeletal elements is certainly partly responsible for the diversity of locomotor mechanics among craniates. The arrangements of the axial musculature among vertebrates show at least as much diversity as any other muscle system. Understanding the adaptive value of the various muscular arrangements is an undertaking to which this review attempts to contribute. To develop a plausible scenario of the evolutionary transitions in the structure and function of the perivertebral musculature, the functional, anatomical, and physiological characters of representatives of pivotal taxa were mapped onto a phylogenetic hypothesis. Such an approach allows inference of the most likely character set of the last common ancestor of the respective taxa as well as informed speculations concerning the patterns of locomotor and muscular evolution. The function of a muscle can be deduced from morphological and physiological variables such as its topography, fiber architecture, fiber-type composition, in-vivo muscle strain and ex-vivo work loops. The integration of these data with other physiological data such as the muscle's activity as well as with biomechanical data such as the associated locomotor kinematics allows one to test functional hypotheses and to infer a muscle's possible functions. Because only some of these variables have been studied in axial muscles of a number of craniates, inference of the muscle function will be based on a subset of this ideally available information (i.e., muscle topography, fiber architecture, fiber-type composition, activation patterns, kinematics).

Parts of the proposed scenario cannot be tested directly because some kinds of information, such as data about soft tissues, are either inadequately preserved in the fossil record or are missing altogether. An indirect method, the 'extant phylogenetic bracket' often allows reconstruction of soft tissue characters of fossils [1]. Hypotheses are thereby formulated by evaluating osteological character states causally related with the tested characters in at least the first two extant outgroups of the fossil taxon of interest [outgroup rule, [2]]. Regarding the axial system, simple inference from extant sister taxa fails in some cases because of the fundamental anatomical differences among the groups and the absence of the critical osteological traits in the respective sister taxa. Additionally, the data available on soft tissue characters such as fiber composition are currently too incomplete for many extant craniates to allow a strict phylogenetic reconstruction of the evolution of their axial system. Assuming that the same biomechanical laws operate now as have in the past, the inferred intramuscular transformations that accompanied gross-anatomical and functional changes during craniate evolution were inferred from studying species that resemble the hypothetical last common ancestor of the particular taxon of interest. For that reason, this review focuses on specific craniate taxa only. Groups highly derived in their postcranial anatomy and locomotor style such as snakes, birds, or monotremes were not included in the proposed scenario; although, of course, they would be potentially interesting and relevant to some of the major themes discussed below.

Axial muscles may serve a number of different locomotor functions. They may produce movements of the axial skeleton that generate positive or negative external work (referred to as mobilization). They also may counteract, control, or restrict movements that are either passively induced by gravitational or inertial forces, actively produced by antagonists, or transmitted to the trunk by extrinsic limb muscles, i.e. they stabilize the trunk. Such stabilizing role may involve long periods of activation, for example to ensure such as the structural linking of the skeletal elements (called tonic, local stabilization), but also faster, briefer muscle action for quick responses for example required to stabilize the trunk against rapid loading (dynamic, global stabilization). Accordingly, local stabilizers can be expected to contain high proportions of fatigue-resistant fibers and are likely in close proximity to the joint they stabilize, while global stabilizers should contain primarily fast contracting fibers and be well effectively positioned relative to the axis of motion. Mobilization, for example to produce body propulsion, may involve slow or fast fibers depending on locomotor speed. As is the case for global stabilizers, mobilizers are expected to be well situated for the production of locomotor work. This classification, first proposed as human-specific trait based on their back muscle topography and activity [3,4], was adopted and further developed by research on other mammalian species [e.g., [5,6]], and revealed as generally applicable to the trunk musculature of tetrapods [e.g., [7]]. Although too strict categorization risks oversimplification, because muscles likely fulfill different functions during different behaviors or even the course of one behavior, such classification of the perivertebral muscles into local and global stabilizers as well as global mobilizers has heuristic value and provides a framework for the formulation of testable hypotheses [8]. Because the evolution of the axial muscle function and morphology is tightly linked to the evolution of the postcranial skeleton, a few relevant aspects of the evolutionary transformations in the postcranial skeleton will first be summarized before the evolution of the perivertebral musculature is discussed.

## Evolution of the postcranial skeleton

The evolution in the postcranial system in craniates from the agnathan fish ancestors to mammals is characterized by two major transformations. First, the axial skeleton became more and more regionalized. Second, the ancestrally axial-based locomotion became increasingly appendage-based with the evolution of extremities and their reorganization within tetrapods. Both events were associated with fundamental changes in the body planes in which movements occur and moments act on the body. Furthermore, the moments acting on the trunk changed substantially during tetrapod evolution with the transition to land.

**Figure 1:**
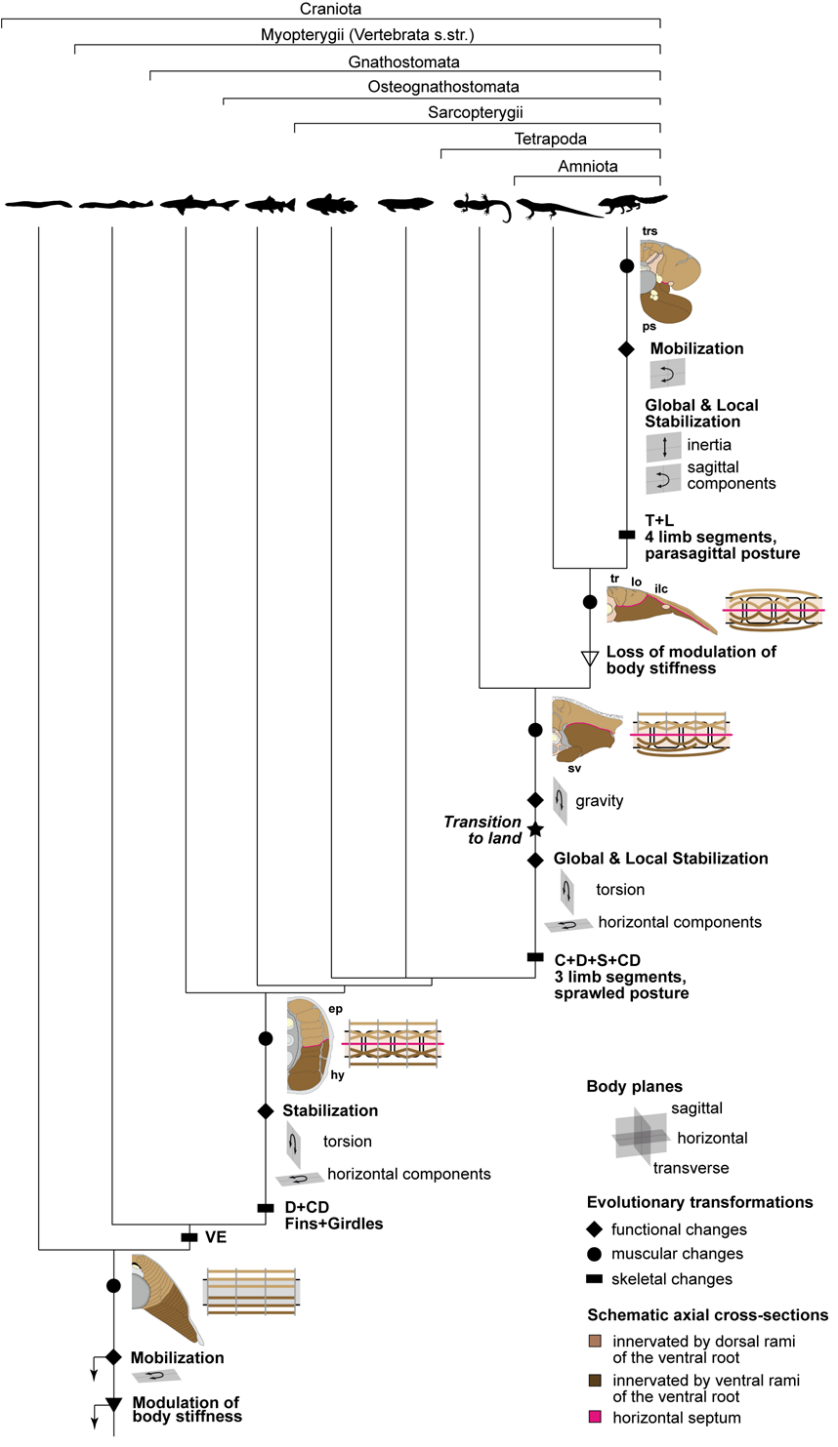
**Hypothesized evolutionary transformations of the morphology and function of the axial system in craniates**. Data were compiled from various sources (see text) and mapped onto a simplified phylogenetic hypothesis based on [71]. Character states plesiomorphic for craniates are indicated by arrows. -- **Axial skeleton **(rectangles): Notochordates (i.e., Cephalochordata + Craniata) ancestrally possess a notochord, eponymous for the group. In early vertebrates, cranio-caudally uniform vertebral elements evolved (VE). In gnathostomes, the axial skeleton is regionalized. A trunk (= dorsal, D) and tail region (caudal, CD) are distinguished in gnathostome fishes, while a cervical (C), truncal, sacral (S), and caudal region are present in early tetrapods. In mammals, the truncal region is further subdivided into a thoracic (T) and a lumbar (L) region. -- **Axial musculature **(circles): Gross anatomy and fiber orientation: Transformations in the arrangement of the perivertebral musculature are illustrated by schematic cross-sections showing the gross-anatomical changes (left) and cartoons of a few body segments in lateral perspective illustrating the changes in muscle and/or fiber arrangement (right). Dorsal and ventral parts of the myomeres are innervated by separate rami of the ventral root in agnathan fishes (light and dark brown). In each segment, muscle fibers span longitudinally between adjacent myosepta. In gnathostomes, the dorsal and ventral myomere parts are morphologically separated by the horizontal septum (pink) resulting in epaxial (ep) and hypaxial (hy) muscles. Likely associated with the evolutionarily new requirements to stabilize the body against long-axis torsion, deeper muscle fibers are obliquely oriented. In non-amniote tetrapods, the epaxial musculature retained its segmental organization in contrast to the hypaxial musculature, which comprises the polysegmental subvertebral (sv) and the abdominal wall muscles (the latter are not shown here). The majority of the epaxial fibers connects adjacent myosepta longitudinally, while deeper fibers run at different angles. In amniotes, the epaxial musculature is reorganized into three longitudinal and polysegmental muscle tracts (tr: transversospinal, lo: longissimus, ilc: iliocostalis). In mammals, the transversospinal muscle is subdivided into several entities forming the transversospinal system (trs). The mammalian ventrovertebral musculature is strengthened by the psoas major (ps). -- **Axial muscle function **(diamonds): The plesiomorphic function of the axial musculature is to mobilize the body in the horizontal plane. The horizontal and torsional moments that result from the evolution of fins and a heterocercal tail, which tend to laterally bend the trunk and cause long-axis torsion, respectively, have to be counteracted by the axial muscles in gnathostome fishes. In tetrapods, as a consequence of the evolution of supporting limbs and transition to land, the axial muscles additionally function to globally stabilize the trunk against inertial and extrinsic limb muscle forces as well as against gravitational forces. Note that the evolution of limbs preceded the transition to land. In tetrapods with a sprawled limb posture, extrinsic limb muscle forces in the horizontal plane are relatively large. The greater agility and maneuverability as well as an increased importance of limb action for body propulsion, likely requires the axial muscles to dynamically stabilize the trunk to a greater extent in amniotes than in non-amniote tetrapods. Associated with the evolution of sagittal mobility and a parasagittal limb posture in mammals, the axial muscles additionally function to globally stabilize the trunk against sagittal components of extrinsic limb muscle action as well as against inertia. Furthermore, the axial musculature mobilizes the trunk in the sagittal plane during asymmetrical gaits.

In petromyzontids, the axial skeleton consists of more or less similar, arch shaped elements situated dorsally to the notochord (arcualia) (Figure 1). In gnathostome fishes, the vertebral column is regionalized into trunk and tail by the presence of ribs and large neural and hemal spines, whereas cervical, truncal, sacral, and tail regions are distinguished in tetrapods. In mammals, the truncal series was further subdivided into a thoracic and a lumbar region, resulting in altogether five morphologically and functionally different divisions of the vertebral column (Figure 1).

Subdivisions of the axial skeleton allow particular body regions to be morphologically and physiologically specialized for certain functions such as body propulsion. For example, the primary function of the tail in gnathostome fishes is propelling the body by lateral undulations [e.g., carangiform swimming, [9]] and therefore it must allow lateral flexion but resist longitudinal compression. In adaptation to this locomotor function, the tail region has no ribs and large hemal arches to provide attachment sites and leverage for the axial muscles. This cranio-caudal regionalization of the body is augmented by soft tissue traits such as differences in fiber population [10,11], fiber contractile properties [12,13] or the arrangement of the connective tissue [14-16]. The reduction of the role of trunk bending in locomotion in carangiform swimmers compared to anguilliform locomotion, as for example in agnathans, may reduce pressure peaks in the body cavity, and thereby interference with inner organ function, but first and foremost it reduces the internal work of locomotion because only part of the body undergoes bending. Similarly, the formation of functional regions of the mammalian trunk facilitated specialization of the vertebral series. The thoracic region allows movements in the horizontal and transverse planes, reflected by more or less horizontally oriented zygapophyses, and the presence of ribs forming the rib cage provides rigidity for the thorax to ensure lung function [17-20]. In contrast, intense motions in the sagittal plane are facilitated in the rib-free lumbar region due to vertical zygapophyses [19-21].

In contrast to the primarily axial-based locomotion of aquatic craniates, body propulsion results from integrated action of trunk and limbs in tetrapods. Therefore, in addition to the plesiomorphic function of contributing to the work of locomotion, the body axis provides the foundation for the production of mechanical work by the limbs, and thus is central to the static and dynamic control of body posture and the integration of coordinated actions of the limbs in all tetrapods. In the lineage leading from the hypothesized ancestor of tetrapods to therian mammals, body propulsion became increasingly dependent on limb action. In salamanders and lizards, the fore- and hindlimbs are composed of three serially homologous elements that function roughly in the same manner regarding their range of excursion and positioning during locomotion [22]. The evolutionary transformation from the ancestral (tetrapod) sprawled limb posture to the derived parasagittal position in therian mammals entailed a dissociation of serial and functional homologues [23,24]. With the reduction of the coracoid, the scapula lost its rigid connection to the trunk in therian mammals and gained mobility unique among tetrapods. In the hindlimb, the proximal part of the autopodium was elongated to form a new functional segment. As a result, the typical therian limb consists of three functionally equivalent elements plus a contact segment [i.e., scapula-femur, humerus-shank, lower arm-metatarsus, hand-toes [23,25]]. Associated with the evolution of a parasagittal limb posture was a fundamental change in the moments that act on the trunk. While extrinsic pro- and retractor muscle activity can be expected to act primarily in the horizontal plane and thus cause lateral bending in a sprawled limb posture, swinging the legs back and forth in a parasagittal plane results in the limb pro- and retractors acting on the trunk in the sagittal plane and thus causing sagittal bending [26]. Furthermore, the lateral components of the propulsive forces, that tend to laterally bend the trunk and exert rotational torque on the girdles, are larger in an animal with a sprawled limb posture compared to one with parasagittal limb motion [27]*vs*. [28].

## Evolution of axial muscle function and morphology

### Agnathans

**Figure 2:**
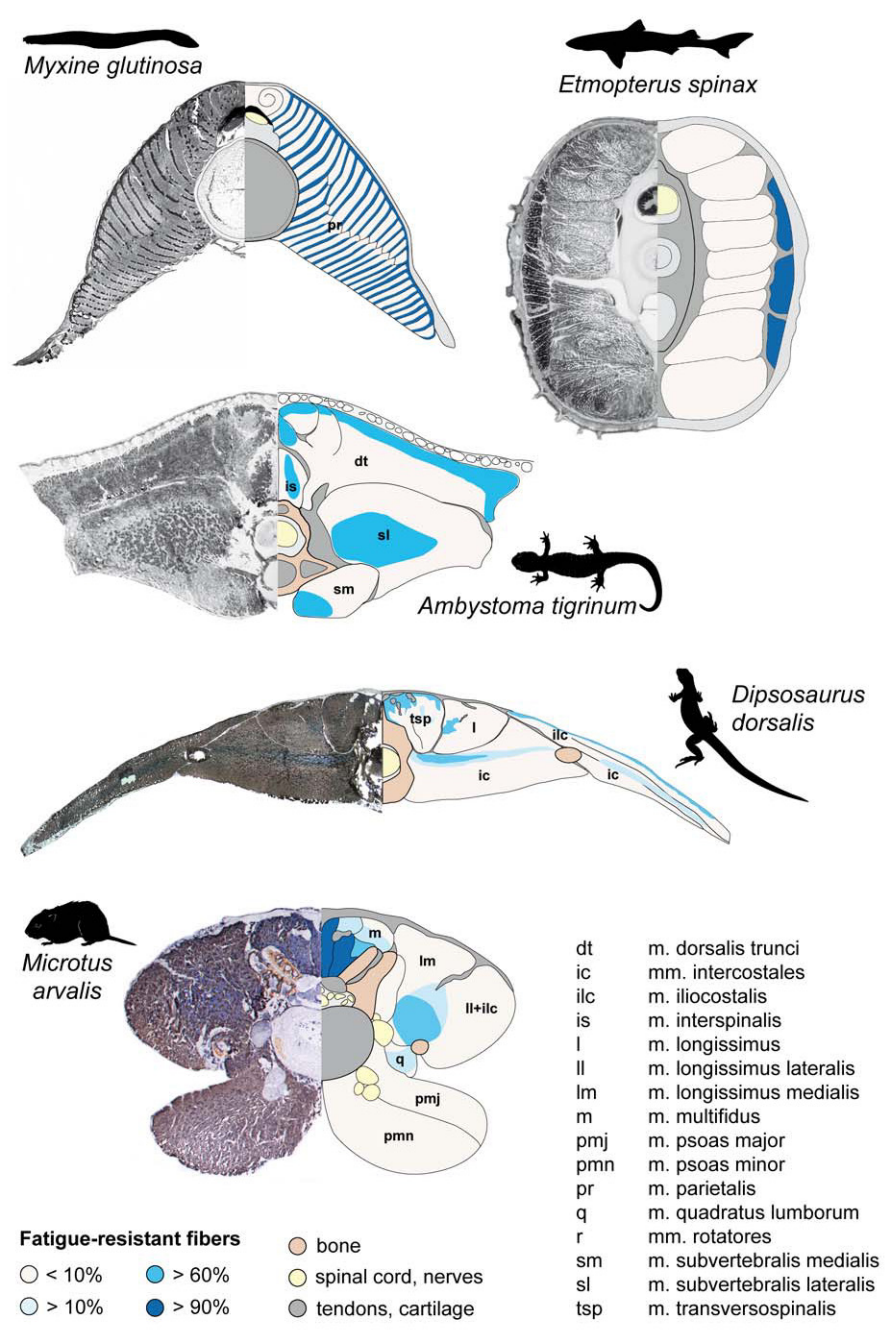
**Histological cross-sections of the perivertebral musculature showing the distribution of the muscle fiber types (left) and schematic illustration of the segregations of fatigue-resistant fibers (right)**. Data were assembled from: hagfish, *Myxine glutinosa*: Sudan black B staining [from [30], reproduced with permission of author and Springer Verlag]; velvet belly lantern shark, *Etmopterus spinax*: cross-section from behind the anus, Sudan black B staining (Photos by P.R. Flood, Copyright by Bathybiologica AS); tiger salamander, *Ambystoma tigrinum*, 4th external trunk segment, enzyme-histochemical reaction for mATPase (acid preincubation) [7]; desert iguana, *Dipsosaurus dorsalis*, 14th trunk vertebra, combined enzyme-histochemical reaction for mATPase (alkaline preincubation) and NADH-TR (S. Moritz, unpubl. data); common vole, *Microtus arvalis*, intervertebral level between 6th and 7th lumbar vertebrae, enzyme-histochemical reaction for mATPase (alkaline preincubation) and NADH-TR [8]. Cross-sections were selected to illustrate of the muscular characters discussed in the text. Note that cranio-caudal changes in the proportion of the respective fiber types may occur (see text for details).

The organization of the axial musculature into serial units (i.e., myomeres) by a complex myoseptal system is plesiomorphic for craniates (Figures. 1, 2). Each myomere is composed of a superficial layer of tonic fibers and a central stack of twitch fibers, all fibers spanning longitudinally between adjacent myosepta [29-35]. The dorsal part of the myomere is innervated by a dorsal branch of the ventral root, while the ventral portion is innervated by a ventral branch [36]. These two myomere portions are innervated by different motoneurons [37], intermingled in the ventral portion of the gray matter of the spinal chord [38]. Each motoneuron innervates muscle fibers in two or three myomeres, resulting in contractions that extend beyond a given segment [36].

Observations on swimming lampreys show a rhythmic, alternating, and posteriorly propagating activation of the axial musculature suitable for producing a traveling wave of lateral bending [39,40]. In both, hagfish and lampreys, the whole body is involved in the undulatory movements with little longitudinal variation in either the burst duration as a percentage of cycle duration or in the lateral displacement [40,41] (i.e., anguilliform swimming), which likely accounts for the anterior-posteriorly undifferentiated musculoskeletal system; the body segments are a repetition of virtually identical subunits. The generated force is primarily transmitted to the notochord by the myoseptal system. The notochord occupies a position near the neutral axis of lateral bending and has been shown to 1) dominate the viscoelastic properties of the body, 2) provide dynamic passive stability, and 3) act as a power amplifier in hagfish [42,43]. It has been suggested that the muscular system actively tunes the body's stiffness in order to match its resonant frequency to undulatory frequency during locomotion [42-44]. Particularly the superficial, tonic fibers are well suited to modulate the stiffness of the body over long periods; possibly directly *via *the myoseptal system and indirectly *via *the skin, onto which the myosepta attach [45]. The parietal, tonic fibers could also be involved in slow frequency swimming, as has been shown for various gnathostome fishes (see below), but unfortunately, no separate recordings from the parietal *vs*. the central fibers exist.

Given the great similarities in myotome organization between lancelets and agnathan craniates [29,38,46,47], the morphology and the function of the axial musculature of agnathan craniates to 1) produce lateral bending, and thus to mobilize the trunk, and 2) to modulate the body's stiffness are most likely plesiomorphic for craniates (Figure 1).

### Gnathostome fishes

In contrast to agnathan fishes and lancelets, a transverse septum (*Septum horizontale*) separates the myomeres into epaxial and hypaxial parts in gnathostomes, which are innervated by separate rami of the ventral root of the spinal nerve. This general separation into epaxial and hypaxial muscles is retained in all gnathostomes, regardless of how profoundly the axial musculature was reorganized in the different taxa. The traditional view of epaxial and hypaxial muscles with their respective innervation is challenged however by the fact that dorsal and ventral parts of the myomeres are also innervated by separate rami in the hagfish [Peters, 1963, cited in [38]] and the lamprey [37]. Therefore, the horizontal septum morphologically separates two previously neurologically distinct units in gnathostomes [48]. Further, in actinopterygian and lungfishes three rami emerging from the ventral root innervate the dorsal, medial, and ventral parts of a myomere, respectively [48-50]. Most likely associated with that, the extreme dorsal and ventral portions show distinct activation patterns that are not necessarily correlated with the activity of the central fibers near the horizontal septum [51]. Nevertheless, the horizontal septum represents the major transmitter of muscle force to the axial skeleton [52], and therefore represents an important locomotor adaptation apomorphic for gnathostome fishes (Figure 1).

Gnathostome fishes have complexly folded, W-shaped myomeres [45,48,53], which are primarily composed of twitch fibers. Tonic fibers are segregated superficially and laterally in a wedge-shaped area close to the horizontal septum (Figure 2), providing good leverage for the production of lateral bending [e.g., chondrichthyans: [54,55]; actinopterygians: [47,56]; lungfish: [10]]. Considerable variation in the amount of tonic fibers and the relative proportion of tonic to twitch fibers may occur along the body or interspecifically and depending on lifestyle [e.g., [10,57-59]], but the general arrangement is very similar among gnathostome fishes. In chondrichthyan fishes, one spinal nerve innervates muscle fibers in two adjacent myomeres [38]. Similar to agnathans, the axial muscles of gnathostome fishes are activated alternating and sequentially consistent with the production of a traveling wave of trunk bending [e.g., chondrichthyan: [60]; actinopterygians: [59,61-63]; lungfish: [64]]. Red, tonic fibers are active during low-tailbeat-frequency, sustained swimming, while white, twitch fibers are additionally recruited during fast bursts and high-tailbeat-frequency swimming [e.g., [65-70]].

The evolution of paired extremities increased the maneuverability in gnathostomes [71]. The associated extrinsic muscles apply forces to the body that induce torsional and bending moments on the trunk. Because many gnathostome fishes are neutrally buoyant, primarily the horizontal (fore/aft) and the lateral components of the propulsive forces produced by the fins play a role in locomotion. The horizontal components cause rotational torque on the girdles and thus lateral bending, requiring preferably longitudinal fiber orientation for stabilization, while the lateral components induce long-axis torsion and require an oblique fiber orientation [26,72]. Because early representatives of gnathostomes such as placoderms typically had a heterocercal tail fin, additional torque about the long-axis of the body likely resulted from tail beating. Compared to agnathan fishes, in which the muscle fibers are oriented longitudinally [29], the evolutionarily new requirements to stabilize the body against long-axis torsion are reflected by the apomorphic oblique fiber orientation found in most gnathostome fishes. For example, the fibers are parallel to the long axis of the body in the superficial portion of the epaxial myomeres, while deeper fibers run at angles between 10° and 35° relative to the body axis [73]. In the lateral hypaxial musculature, muscle fibers of the two oblique layers have opposing radial orientations [45], well suited to stabilize the body against long-axis torsion (Figure 1). In addition, oblique fiber orientation provides an advantage for shortening velocity due to the greater architectural gear ratio, that is, a greater shortening distance resulting from fiber rotation as a consequence of the constant volume of the segment [74].

Hence, the axial musculature of non-tetrapod gnathostomes retained its plesiomorphic function of mobilizing the body and producing locomotor work. Associated with the evolution of fins and a heterocercal tail, the axial musculature also stabilizes the body against the locomotor forces produced by the extrinsic fin muscles and torsional moments resulting from tail beating (Figure 1). These new functions are reflected by an oblique fiber orientation hypothesized to be apomorphic for gnathostomes.

### Tetrapods

The plesiomorphic segmental organization of the axial musculature underwent stepwise reorganization during the evolution of tetrapods. In salamanders, the only available postural model for early representatives of the tetrapods, the epaxial musculature retained its plesiomorphic segmental arrangement in contrast to the hypaxial muscles. The hypaxial musculature consists of the abdominal wall muscles and a subvertebral muscle mass, which is associated with the ventral aspect of the vertebrae and ribs. Additional to the rectus system, the abdominal wall generally comprises three layers: the external and the internal oblique muscles as well as the transversus muscle. The latter is an apomorphic feature of tetrapods [53] and involved in ventilation [75]. In most urodeles, the lateral hypaxial musculature is secondarily segmentally organized by tendinous inscriptions [76,77] and displays different fiber angles depending on the layer [78,79]. Associated with the evolution of polysegmental hypaxial muscles was likely a change in muscle fiber type distribution from a superficial position of fatigue-resistant fibers in fishes to a deep localization in tetrapods such as salamanders [7]. As in gnathostome fishes and thus plesiomorphic for tetrapods, the majority of the fibers connect adjacent myosepta longitudinally; only deeper fibers associated with the vertebrae run at different angles within the epaxial myomeres [80-82]. The segregation of the muscle fiber types in the epaxial musculature of urodeles resembles the pattern plesiomorphic for craniates [47,83]. That is, tonic and slow-twitch fibers are co-localized superficially, while fast-twitch fibers form the bulk of the deep muscle [7,84] (Figure 2). In the only two salamander species for which data exist so far, this pattern is more or less unchanged along the trunk [7].

Similar to fishes, when salamanders swim, their main epaxial and all hypaxial muscles are active synchronously and alternating. Activation propagates along the body, consistent in timing with the production of a traveling wave of lateral undulation [85-90]. Thus, in salamanders, most axial muscles mobilize the body during swimming, i.e. their plesiomorphic function is retained. In accordance with its poor mechanical advantage for trunk bending and high percentage of tonic red and twitch intermediate muscle fibers [7], the biphasic activity of the interspinalis muscle suggests that this muscle functions in vertebral stabilization rather than lateral bending [90]. Active modulation of the body's stiffness was suggested as one of the adaptations to swimming in salamanders [85], and the superficial segregation of fatigue-resistant fibers in the dorsalis trunci muscle could modulate the body stiffness *via *the myoseptal system and the skin [7]. Unfortunately, no study has investigated the recruitment patterns of the different fiber populations in this muscle, but the striking resemblance of myomere organization to non-tetrapod craniates invites such speculation. Nevertheless, when salamanders swim, most of their axial muscles produce lateral bending, some likely also modulate the body's stiffness, and others provide local stabilization.

The evolution of limbs predated the transition to land as has been argued based on the analysis of early representatives of tetrapods such as *Acanthostega *[91] and members of the sister-group of tetrapods such as *Tiktaalik *[92]. Because aquatic stepping was likely the primitive locomotor function of the tetrapod limb [93], trunk stabilization against locomotor forces produced by extrinsic limb muscles is evolutionarily older than stabilization against gravitational forces. Thus, the evolutionary transition to land, basically a transition from high to low viscosity and density and from low to high gravitational loads, was primarily associated with decreased inertia and drag during the limb's swing phase and increased gravitational loading of the body resulting in increased postural work for limb and trunk muscles [94]. Furthermore, the vertical components of the forces produced by the limbs, that are partially compensated by buoyancy during aquatic stepping, induce long-axis torsion of the body during terrestrial stepping [26].

A comparison of axial muscle activity during aquatic and terrestrial stepping showed that muscle recruitment (i.e., intensity) increased in all trunk muscles, despite similar temporal patterns of muscle activation [90]. This suggests that the trunk is stiffened during terrestrial locomotion, whereas the basic functions of the muscles are conserved across environments. Consistent with this, the perivertebral musculature contains an overall higher proportion of red tonic and intermediate twitch fibers in salamanders when compared to other sarcopterygians such as lungfish. Comparisons of the fiber type composition in various ecotypes, for example of predominantly terrestrial *vs*. aquatic species would allow testing this hypothesis. In addition, fatigue-resistant fibers are segregated in a central region of the lateral part and in ventral proximity to the vertebral column in the medial part of the subvertebral muscle, allowing them to provide stability against torsion and sagging, respectively [7].

**Figure 3:**
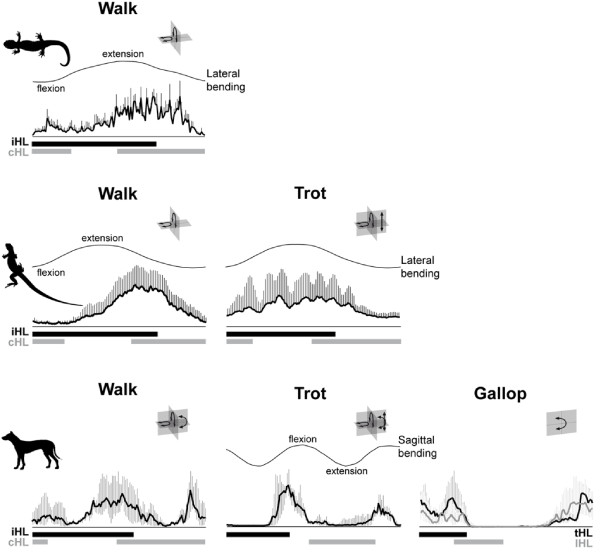
**Activity patterns and hypothesized functions of the epaxial muscles in tetrapods during locomotion [modified from **[118]]. Data for the epaxial muscle activity were assembled from: spotted salamander, *Ambystoma maculatum*, m. dorsalis trunci, 8th external trunk segment, mean and standard error [90]; desert iguana, *Dipsosaurus dorsalis*, m. longissimus dorsi, 14th trunk vertebra, mean and standard deviation (S. Moritz, unpubl. data); dog, *Canis familiaris*, m. longissimus thoracis et lumborum, 6th lumbar vertebra, median and upper and lower quartiles [118]. The x-axis represents the stride cycle beginning with the touch down of the ipsilateral hindlimb. The footfall patterns of the both hindlimbs are illustrated on the bottom of each graph (walk, trot: black: ipsilateral limb (iHL), gray: contralateral limb (cHL); gallop: black: trailing limb (tHL), gray: leading limb (lHL). Note that for the galloping dog, the EMG trace associated with the trailing hindlimb is black, the one associated with the leading hindlimb is gray. Bending traces above the electromyograms indicate the unimodal lateral flexion and extension on the body side ipsilateral to the recorded muscle activity (salamander, lizard) and the bimodal flexion and extension in the sagittal plane (mammal). Body planes in which moments and/or movements are suggested to occur are illustrated in the right top corner of each graph (for details see Figure 1). Note that the unilateral and monophasic epaxial activity in the walking salamander and lizard associated with the ipsilateral stance phase corresponds to the main activity observed in mammals. In mammals, the increased need for sagittal stability is met by bilateral activity resulting from a second burst during ipsilateral swing phase.

During both aquatic and terrestrial stepping, body propulsion is achieved by concerted trunk and limb muscle action in salamanders. Lateral bending was suggested to be actively produced by the trunk muscles to facilitate the placement of the feet, which serve as anchors and contribute to stride length [95,96]. But lateral bending may also result passively from extrinsic limb muscle action acting on the trunk *via *the limb girdles [97,98]. Consistent with the production of a standing wave of lateral bending, uniphasic and cranio-caudally synchronized activity of the majority of the trunk muscles has been observed [85-90] (Figure 3). Additional bursts close to limb girdles indicate that the dorsalis trunci muscle also stabilizes the trunk against limb muscle action [88]. This additional activity likely serves to dynamically stabilize the trunk in the horizontal plane. Accordingly, the muscle primarily contains white twitch fibers [7,99], which are arranged parallel to the long-axis of the body [80,81,100] and the fore/aft and lateral components of extrinsic limb muscle action can be expected to be greater than the vertical ones given the sprawled limb posture. Consistent with their oblique fiber orientation [77,80], activity of the lateral hypaxial muscles resists long-axis torsion [86,89,90]. The biphasic activity of the fatigue-resistant interspinalis muscle suggests that it functions as a local stabilizer during stepping, similar to its function during swimming [90].

In summary, the axial musculature of basal tetrapods such as salamanders mobilizes the trunk by producing lateral bending, modulates body stiffness (both putative plesiomorphic) and provides local stability to ensure the integrity of the axial skeleton during swimming (putative apomorphic for tetrapods). During aquatic stepping, it additionally resists extrinsic limb muscle forces causing lateral bending and long-axis torsion of the trunk; functions likely plesiomorphic for the group. During terrestrial locomotion, the axial musculature also stabilizes the body against gravitational forces (Figure 1); an apomorphic function for terrestrial tetrapods.

### Amniotes

A notable difference between anamniote and amniote tetrapods is the greater terrestrial agility in amniotes. Early amniotes were gracile, small animals with a snout-vent length of up to 24 cm [e.g., *Paleothyris *or *Hylonomus*, [101]], and thus comparable to extant small lizards such as desert iguanas. Analyses of the axial skeleton and reconstructions of the associated musculature in various fossils indicate great similarity between these early amniotes and generalized extant lizards and therefore imply similar trunk motions [102,103]. Their diet and associated with that their lifestyle was presumably also similar to extant small lizards, i.e. mainly preying on arthropods, mixed with some plant material [104,105]. Therefore, both burst and slow locomotion must have constituted the locomotor repertoire of early amniotes. Associated with a higher aerobic capacity [106] and relatively higher body temperatures during activity [107], amniotes such as lizards are characterized by greater swiftness and maneuverability compared to anamniote tetrapods such as salamanders. Swifter movements and increased performance are connected with faster accelerations and decelerations of the limbs and the center of mass of the body (CoM), and thus higher peak loading of the limbs and trunk. Consequently, amniotes have an increased need for dynamic stabilization of the body compared to anamniote tetrapods.

Similar to lissamphibians, amniotes such as lizards exhibit a sprawling limb posture in which the feet are positioned far laterally from the body axis. Compared to a parasagittal limb posture, a sprawling posture is associated with greater lateral components of the propulsive forces see [[27]*vs*. [28]] and greater horizontal components of extrinsic limb muscle forces due to pro- and retraction of the stylopods in the horizontal plane [22,26,108,109]. Both aspects result in moments that laterally bend the trunk. Compared to salamanders, limb action can be expected to play a greater role in the production of locomotor work of lizards because of their relatively stronger limbs and greater limb excursions. Therefore, lateral bending in lizards may be a consequence of limb posture and limb muscle action, in addition to being actively produced for example to contribute to the production of locomotor work [72] and to facilitate limb positioning [109,110]. It is hypothesized that during the evolution of amniotes a shift in trunk muscle function occurred from primarily producing lateral bending (anamniote tetrapod mode) to increasingly controlling and counteracting moments caused by limb action and greater peak loading.

In amniotes, the epaxial muscle mass was reorganized into longitudinal and polysegmental tracts, forming the transversospinal, the longissimus, and the iliocostalis groups (Figure 2). The complexity and the arrangement of these tracts vary greatly among amniotes due to differentiation into smaller muscle units and/or variations in their relative sizes [20,100]. The hypaxial musculature shows a wide range of variation in ectothermic amniotes such as lizards primarily due to splitting and delamination of the main layers [111,112]. This anatomically more complex arrangement compared to other tetrapods such as salamanders is likely partially related to their enhanced locomotor performance but likely also because axial muscles fulfill other functions such as ventilation in addition to their plesiomorphic role in locomotion [113]. As in salamanders, the muscle fibers in the various layers of the lateral hypaxial musculature are oriented obliquely at different angles [72,111]. In the epaxial musculature, the most medial tract shows an oblique fiber orientation in lizards, while the fibers in the two lateral tracts are more or less parallel to the long-axis of the body [100,114,115]. In contrast to anamniotes, in which the motoneuron pools of the epaxial and hypaxial muscles overlap in the medial column, motoneurons are spatially segregated in amniotes [116]. Motoneurons innervating epaxial muscles are located in the ventromedial portion of the ventral horn, while the hypaxial motoneurons reside dorsolaterally. Therefore, discrete pools serve individual muscles, resulting in a topographic map of motor pool organization that likely facilitates proper control of the anatomically and, more importantly, functionally diverse muscles originating from the same myotome [38].

It remains controversial whether or not epaxial and hypaxial muscles are involved in the production or the counteraction of lateral bending in lizards as they are in salamanders [117,118]. A functional division between epaxial and lateral hypaxial muscles was proposed as a basal feature of amniotes [117]; the former serving to stabilize the trunk against torsional forces [119], while the latter function to laterally bend the trunk and provide stabilization against long-axis torsion [72]. For the epaxial muscles, Ritter concluded that they are not involved in bending based on the timing of the activity as well as denervation experiments [117]. Several observations question this hypothesis: 1) Recent recordings from walking lizards do suggest that the timing of the activity of the epaxial muscles is consistent with the production of lateral bending [120] (Figure 3) and thereby confirm previous recordings [110]. These recent data imply speed dependency in the epaxial muscle function, and thus may reconcile the controversy observations [120]. 2) The denervation experiment, which provided the main evidence against lateral stabilization, was carried out around the mid-trunk, where the impact of the extrinsic limb muscles is likely to be small. Also, possible compensatory actions of other muscles such as the hypaxial muscles were not tested. Furthermore, the timing of epaxial muscle activity in lizards is similar to that in salamanders and mammals, for which a stabilizing function against lateral bending was shown, at least near the limb girdles, by simultaneous recordings of extrinsic limb and back muscles [88,121]. 3) The importance of lateral trunk bending, its production or counteraction, is reflected in the anatomy of the epaxial muscles. The two lateral tracts, well positioned to act laterally on the vertebral column, are relatively large in lizards [122], and their muscle fibers are oriented longitudinally, a fiber orientation well suited to laterally mobilize and stabilize the trunk [100,114,115]. Thus, a mobilizing and/or stabilizing role in lateral bending cannot be ruled out for the epaxial muscles in lizards and further experiments, for example manipulating the locomotor forces, are necessary to clarify the function of the epaxial muscles in lizards.

In addition to the plesiomorphic side-to-side movements, rotations about the long-axis of the body are an important component of amniote locomotion and particularly the transversospinalis muscle was thought to provide torsional stabilization based on its activity [117,119] and the morphology of the neural spines [103]. Its oblique fiber orientation [100,114] is consistent with a stabilizing function against long-axis torsion and distinguishes amniote from anamniote tetrapods. As pointed out above, compared to salamanders, extrinsic limb muscle and inertial forces can be expected to be greater in lizards with their greater agility and locomotor speed. Therefore, torsional stabilization is additionally provided by the epaxial musculature of lizards [117], but solely accomplished by the lateral hypaxial muscles in salamanders [86,89].

The evolutionary disintegration of the plesiomorphic segmental organization of the epaxial musculature of tetrapods resulted in longitudinal, polysegmental muscle tracts in amniotes and, likely more importantly, in an overlapping muscle arrangement. Although this segmental disintegration may be connected with a slightly increased number of sarcomeres in series and thereby a small increase in contraction speed, one advantage of a polysegmental over a segmental arrangement may be that it allows for stabilization or mobilization of a whole region of the trunk by activating a single motor unit. In contrast, simultaneous activation of several adjacent segments is required in a myomeric organization to affect a larger body region (e.g., to produce a standing wave). Simultaneous action on a body region may be advantageous if the primary mode of trunk bending during locomotion is a standing wave, rather than a traveling wave, during which adjacent segments undergo lateral excursion sequentially. On the other hand, an overlapping arrangement with attachment sites on each vertebra also allows the production of a traveling wave, as for example in snakes [123,124]. But, more importantly, the possession of muscle fibers of different lengths organized in an overlapping arrangement may increase the animal's maneuverability because it allows for activation and control of specific and varying body regions and thus for greater versatility. Associated with the reduction of the myoseptal system, the muscle fibers also act directly on the vertebrae in amniotes rather than indirectly *via *the myosepta. Direct muscle action on the vertebral column was associated with a greater degree of vertebral structuring, i.e., relatively longer processes and larger protuberances, which provide increased lever arms and attachment sites for the muscles [103,112]. In summary, possibly greater contraction speed and distance, more precise and selective activation and control of a specific body region due to an overlapping muscle arrangement, and improved muscle lever arms may have facilitated more rapid mobilization and stabilization of the body and are likely connected with the greater agility and versatility of amniotes.

Preliminary results on the perivertebral musculature of lizards indicate, when compared with results on mammals [8], similarities in the overall fiber type distribution among these amniotes [125]. Fatigue-resistant fibers are segregated in deeper muscle areas, close to tendons and bones, while the majority of the muscles comprises primarily fast twitch fibers (Figure 2). Consistent with the superficial and polysegmental muscles functioning in mobilization and global stabilization in lizards, they contain primarily fast-twitch glycolytic muscle fibers [125]. To allow these polysegmental muscles to act on a given division of the vertebral column without causing vertebral dislocation, monosegmental muscle fibers are hypothesized to ensure spinal integrity. The demands for local stabilization can be expected to be greater in lizards compared to salamanders due to their greater trunk loading and their polysegmental structure of both epaxial and hypaxial muscles. Given their topography and fatigue-resistant properties [125], local stabilization is probably accomplished by the deeper fibers of the transversospinalis muscle (Figure 2). Unfortunately, no EMG recordings exist of this muscle region to test this hypothesis. In contrast to anamniote tetrapods, in which tonic and slow-twitch fibers are segregated superficially, likely to modulate the body stiffness *via *the myoseptal system and the skin, the fatigue-resistant fibers of amniotes are regionalized in the depth of the muscles close to the bones and intramuscular tendons in amniotes [8] (Figure 2). This intramuscular reorganization has been suggested to be related to the complete independence from water [35]. Independence from water required changes in skin anatomy to reduce evaporation and may have simultaneously decreased the skin's ability to participate in force transmission. Furthermore, the intimate connection between the myoseptal system and the skin was dissolved with the evolution of longitudinal muscle tracts. Thus, axial muscle forces are directly transmitted to the vertebrae in amniotes [112] and the body does not function as a hydrostatic system in body support as in anamniotes. Thus, the loss of the superficial fatigue-resistant fibers may be associated with the substantial reorganization of the epaxial musculature and the high degree of amniote terrestriality.

In summary, the axial musculature of lizards appears to fulfill similar functions as to those in salamanders, allowing tentative inference that these functions are plesiomorphic for amniotes. But, compared to salamanders, the need for local and especially global stabilization of the trunk is increased in lizards due to their greater agility and locomotor speed, and this need is reflected in the detailed muscle morphology.

### Mammals

One of the most striking apomorphic characteristics of mammalian locomotion is sagittal bending [126-128]. The ability to dorsoventrally flex and extend the body axis enabled the evolution of asymmetrical gaits in mammals such as gallop or half-bound [19] [note the convergent evolution of galloping in crocodilians [129-131]]. Several vertebral characteristics have been proposed to be prerequisite for sagittal bending and, thus, to have predictive value for the trunk region involved: 1) reduction of ribs in the posterior trunk and thus the formation of a lumbar region; 2) orientation and width of the spinous processes and thus the position of the anticlinal vertebra in the vertebral series; 3) orientation of the zygapophyseal facets and thus the location of the diaphragmatic vertebra(e) along the vertebral column [18-21,132]. A comparative analysis of intervertebral movements in small therians during fast locomotion showed however that these skeletal characters were not simply related to the trunk region involved in bending during locomotion, questioning their predictive value for the trunk region involved in sagittal bending [133,134]. It has been suggested, therefore, that behaviors other than those directly related to locomotion may have driven the evolution of sagittal mobility, which was subsequently incorporated into the locomotor repertoire [133,135].

During the evolution of mammals, extensive fusion and reorganization of the epaxial tracts was associated with the reduction of the posterior ribs and the evolution of a rib-free lumbar region. In many mammals, the two lateral tracts are inseparable in the lumbar region and therefore referred to as the sacrospinalis muscle [100]. Associated with the evolution of asymmetrical gaits and the corresponding intense dorsoventral flexion of the posterior trunk were major reorganizations in the muscular system. For example, in mammals the size of the two medial epaxial tracts is increased compared to lizards [20]. Accordingly, both the multifidus and the longissimus muscles exhibit recruitment patterns consistent with the mobilization of the axial skeleton in galloping mammals [118,136,137]. Additionally, compared to non-mammalian amniotes, the subvertebral musculature was strengthened in mammals and assists the abdominal wall muscles as an antagonist of the epaxial musculature. Parts of the hindlimb musculature shifted onto the trunk (i.e., puboischiofemoralis as iliopsoas muscle) and an axial slip of the subvertebralis muscle became independent as the psoas minor muscle [112,138,139]. Both act as hindlimb protractors and flexors of the vertebral column in mammals [140-143]. These muscular changes in the epaxial and the hypaxial musculature augmented the fast, glycolytic muscle mass around the vertebral column and therefore were likely associated with the evolution of vigorous sagittal spine movements, i.e. the evolution of asymmetrical gaits [8]. Consistent with the caudally increasing importance of sagittal bending in body propulsion [133], the proportion of glycolytic muscle mass relative to the total anatomical cross-sectional area of the axial musculature increases caudally [5,6,8].

The increased mobility in the posterior trunk and its vigorous mobilization during fast locomotor activities was hypothesized to be associated with an increased need for local stabilization [8]. The evolutionary subdivision of the transversospinalis muscle into several muscular entities in mammals (i.e., the transversospinal system) is probably related to this greater demand for intervertebral stabilization because it was accompanied by the functional specialization of its subunits [8]. Several deep, mono- and polysegmental muscles evolved (e.g., rotatores, intermammillares, mammilloaccessorii muscles) and are predominantly composed of fatigue-resistant, slow fibers and thus well suited to provide sustained intervertebral stability [5,6,144]. In contrast, the superficial, multisegmental division of the transversospinal complex (i.e., the multifidus muscle) contains a high proportion of fast fibers [e.g., [8,145,146]; Figure 2] that can mobilize as well as dynamically stabilize the trunk [118,121,137,136].

Another consequence of the greater mobility in all body planes, particularly in the posterior trunk, is an increased need for postural feedback. Mammals differ from other amniotes in that they possess a central, slow region in the in the lateral longissimus muscle, which extends between the iliac blades and the 4th to 2nd presacral vertebrae [8]. This region contains a large number of muscles spindles [147,148] and is activated tonically and independently from the rest of the muscle belly [149,150]. Its responsiveness is modulated by the vertebral position [151]. It was suggested to function as a proprioceptive system monitoring the position of the pelvis relative to the vertebral column [147,149]. Because no such region has been found in lizards (S. Moritz, pers. commun.) and salamanders [7], it is hypothesized to represent an apomorphic character of mammals and to be correlated with the evolution of a mobile lumbar region [8].

During the evolution of mammals, truncal motions in the sagittal plane were added to the plesiomorphic movements in the horizontal and transverse planes. Both, lateral bending and long-axis torsion occur during symmetrical gaits [e.g., [134,152-155]]. They are, however, less pronounced in mammals than in other tetrapods. The functional roles of the axial muscles during symmetrical gaits have been investigated in more detail in mammals than in any other tetrapod group, but still seem poorly understood compared to the understanding of the limb musculature. Whereas the functional roles of the lateral hypaxial muscles were clarified in a series of experiments [156-158], the function of the epaxial muscles have become more clear only recently. Because their activity was not directly correlated with the production of lateral bending or tilting, the epaxials were suggested to stabilize the trunk [137,159-163]; thereby, only two studies tested the specific locomotor forces and moments that may require stabilization ['sagittal rebound', [164,165]]. Their primary function, at least near the hindlimb girdle, is to provide global stabilization against the vertical components of retractor muscles and the horizontal components of pro- and retractor muscles [121]. Furthermore, epaxial muscles probably assist in the production of lateral bending during symmetrical gaits because the observed cranio-caudal activation patterns during walking and trotting accord in timing with both the traveling and the standing wave of trunk bending observed in these gaits, respectively [118]. Consistent with a function as dynamic stabilizers as well as mobilizers, the largest epaxial muscles (i.e., the multifidus and the sacrospinalis muscles) consist predominantly of fast, glycolytic fibers [see [8] and references therein] (Figure 2).

Compared to the sprawled limb posture of lower tetrapods, the parasagittal limb posture of mammals can be expected to result in relatively smaller lateral but greater sagittal components of the propulsive forces produced by the limbs [97]. Furthermore, although the vertical moments acting on the trunk due to inertia are similar in lizards and mammals with the same size and locomotor speed, they are most likely largely passively stabilized in lizards by their horizontally oriented zygapophyseal facets, but would bend and extend the trunk sagittally in mammals due to their more vertical facets. Both, the locomotor forces produced by the limbs and the inertia of the body, result in an increased need for dynamic muscular stabilization in the sagittal plane. This increased need is reflected by changes in muscle morphology and function in mammals compared to lizards. For example, the two medial epaxials, best suited to provide sagittal stability and mobility due to their more dorsal position relative to the neutral axis of the vertebral column, are increased in size in mammals [20]. Furthermore, all epaxial muscles have a distinct oblique fascicle orientation [100], which allows for mobilization and stabilization in all planes of the body simultaneously and thus better meets the complex needs for trunk mobility and stability in mammals. This oblique fiber orientation likely provides an advantage in the shortening velocity of the entire muscle [74]. Furthermore, all mammals investigated so far display a biphasic and bilateral activity in their epaxial muscles during symmetrical gaits [137,159-166]. Of these two bursts during each locomotor cycle, only the main burst occurring during ipsilateral hindlimb stance corresponds to the epaxial activity observed in other tetrapods (Figure 3), while the second burst, associated with the hindlimb swing phase, distinguishes mammals from other tetrapods [118] and thus appears to be an apomorphic feature of mammals. Based on recruitment symmetry (i.e., bilateral activity) or asymmetry (i.e., unilateral activity) between both body sides a net extensor or net lateral bending/torsional moment can be inferred [167]. A net extensor moment is expected if sagittal forces dominate (e.g., due to the vertical oscillations of the CoM or vertical components of the extrinsic limb muscles), and the main function of the muscle is to stabilize the trunk in the sagittal plane. The fact that mammals consistently show biphasic, bilateral activity in their epaxial muscles corroborates the interpretation that there is an increased need for sagittal stability [118].

Among amniotes, only birds and mammals are able to locomote and ventilate their lungs at the same time [113], except secondarily derived solutions for example in varanid lizards [168]. In mammals, the evolution of a diaphragm freed most axial muscles from a ventilatory function during locomotion [158,169]. Because the diaphragm attaches to the posterior ribs, action of the diaphragm results in anterior tilting of the ribs. To provide a firm base for the action of the diaphragm, the ribs need to be stabilized (e.g., pulled caudally). The abdominal wall muscles, namely the oblique muscles, are well positioned to retract the ribs and counteract rib protraction. However, both the internal and the external obliques are locomotor muscles [158], stabilizing the trunk against sagittal shear during locomotion [157], and therefore cannot provide costal stabilization. Especially during asymmetrical gaits, inhalation is coupled with trunk extension [133,170], thus the oblique abdominal wall muscles would have to stabilize the ribs during sagittal extension. Such activity of the oblique hypaxial muscles would cause sagittal flexion and thus interfere with the extension of the trunk. Rather, the oblique abdominal wall muscles are in a good position to assist the rectus abdominis muscle, which is the most important spinal flexor and active at the appropriate time [137]. EMG recordings of the external oblique muscle in galloping dogs are consistent with such locomotor function [Deban, Schilling, Carrier, unpubl. data]. However, neither during symmetrical nor during asymmetrical gaits can rib stabilization be provided by the abdominal wall muscles.

Rib stabilization and possibly widening of the pleural cavity during inhalation may be provided by the quadratus lumborum muscle based on its activation pattern as has been shown in rabbits [171]. The homology of this muscle has been subject of controversy [i.e., partially subvertebralis, [172], intercostalis system: intertransversarii muscles, [112], levatores costarum muscles, [173]]. Its innervation either from the dorsal or the ventral rami [174] and the location of its motoneurons in the ventromedial and the lateromedial motor pools [175] implies a mixed origin. However, its anatomical position on the ventral aspects of the centra and insertion onto the most posterior ribs allows the quadratus lumborum muscle to provide costal stabilization without interfering with locomotor events. Its proximity to the vertebral column gives it poor leverage for sagittal flexion and therefore its contribution to sagittal bending can be expected to be low. Consistent with its function in rib stabilization, the quadratus lumborum muscle showed a striking central accumulation of slow fatigue-resistant fibers, particularly in its anterior part in various therians [5,6,8,176]. This central region was hypothesized to act independently from the rest of the muscle belly [8], similar to deep slow regions in anti-gravity muscles [177,178]. In accordance with a function in ventilation, its muscle fiber type composition did not show the physiological adaptations found in other perivertebral muscles with changes in body shape [e.g., in ferrets [179]] or body size [176].

In summary, the evolution of sagittal mobility in mammals added a new body plane, in which movements can be produced but also have to be controlled and counteracted. Thus, the axial muscles in mammals mobilize the trunk in the sagittal plane (apomorphic for mammals), in addition to their plesiomorphic role in bending and twisting (Figure 1). In the epaxial musculature, the increased need for dynamic sagittal stabilization due to the parasagittal limb posture and the vertical zygapophyses was met 1) locally by the evolution of numerous deep, short, fatigue-resistant muscles and 2) globally by a biphasic activity of superficial, polysegmental, fast muscles.

## Concluding remarks

### Intramuscular (re)organization in craniates

Muscular properties such as the distribution of muscle fiber types are primarily determined by a muscle's function and less by phylogeny. During the evolution of craniates, the composition and distribution of fiber types changed profoundly with a general tendency to segregate fatigue-resistant fibers in deeper muscle regions. Various factors have been discussed to account for a certain, 'preferred' location of a given fiber type within a muscle or a muscle group such as heat loss or thermal balance [reviewed in [180]]. Briefly, it is argued that because red muscle tissue has better circulation at rest than white one, a superficial position of red fibers would cause greater heat loss [181], assuming that the environment is cooler than the animal. The thermal balance argument is based on the observation that muscle fibers increase their shortening speed and power as they become warmer, which would be advantageous for deeply located, more insulated fibers. The temperature dependence of these characteristics is essentially similar between red and white fibers [e.g., [182-186]], and therefore would support either fiber type distribution. The comparison of the intramuscular organization among craniates illustrates that red or white fibers may be closer to the core of the body indicating that other factors in addition to heat conservation are relevant to intramuscular organization.

Muscle fibers of different types are either segregated from each other within a muscle or a muscle group or they are intermingled ('salt-and-pepper pattern'). Gathering one fiber type may be advantageous because it unites similar metabolic needs, neural control, and biomechanical properties. For example, red and white fibers differ in their blood supply, in both the course and the branching pattern of the capillary network [e.g., [187-189]] as well as in their capillary to fiber ratio [e.g., [190-195]]. Whether the higher capillary content, and thus a relative higher collagen proportion per muscle area due to the vessel walls accounts for the different biomechanical properties reported for red and white muscle tissue [e.g., [189,196]] or differences in the connective tissue itself, for example in the structure of the endomysial collagen [197-200], is controversial, but a greater potential for elastic energy storage and a higher stiffness was found in red compared to white muscle tissue [199,201]. Thus, congregating fibers of similar metabolic needs may reduce the costs of the formation and maintenance of the supply network and concentrating fibers with similar mechanical properties may reduce intramuscular shear [188,202]. Furthermore, segregation of a specific fiber type allows a muscle region to specialize for a specific function, because the properties of the various fiber types are optimized for different motor tasks [203]. Thus, an accumulation of a specific fiber type indicates that this muscle or muscle region fulfills first and foremost the same function in the same manner. In contrast, a mixed composition of a muscle or muscle region places fibers with different contractile properties in the biomechanically advantageous position. Such arrangement allows the muscle to fulfill the same function in different ways, i.e. by using different fiber types and thus different motor units, for example to accomplish the function with various force, speed, or frequency [204,205].

In addition, the reorganization of the myoseptal system into polysegmental muscle tracts resulted in an architectural problem in amniotes. The evolution of polysegmental muscle tracts likely increased the importance of local stabilization of the intervertebral joints to allow the polysegmental muscles to act on larger but variable units of the vertebral column without causing intervertebral instabilities. To provide local stabilization and prevent vertebral dislocation, short muscle bundles containing fatigue-resistant fibers and interlinking the vertebrae (i.e., monosegmental muscles) must be positioned close to the vertebral column, while the polysegmental muscles are necessarily layered above. Thus, simple architectural constraints additionally influence muscle-fiber-type distribution. Further research is necessary to increase our understanding of why muscle fibers of a given type are localized in particular muscle areas and how the observed patterns of muscular organization evolved.

### Methodological caveats

Muscle is one of the most plastic tissues, which allows the study of adaptations to changing functional demands on the one hand, but requires a thorough selection of the individuals and species studied on the other hand, because interindividual or interspecific variability may mask the investigated traits. Hence, observed differences may represent phylogenetic divergence, functional divergence, and/or effects of environmental factors that differed among the individuals studied (phenotypic plasticity). The species discussed herein were selected based on their resemblance (particularly body size and proportions as well as locomotor mode) to early representatives of higher taxa pivotal for the reconstruction of the evolution of the craniate axial system. The muscular differences observed among them are assumed to correlate with evolutionary changes in function and morphology and that these differences are greater than inter-individual variation.

This approach bears several caveats limiting inference of character states. 1) Depending on the fossil record and the availability of extant species resembling early representatives of a given group in the critical traits, the conclusions are better supported in some groups than others. For example, extant small mammals such as mice, rats, or tree-shrews highly resemble Mesozoic mammals such as *Morganucodon *in their postcranial anatomy [reviewed in [8]] and therefore are well-suited to infer soft tissue characters for early mammals. In contrast, salamanders differ in several essential postcranial characters from early representatives of tetrapods such as *Acanthostega *or *Ichthyostega *[91,206] such as the reduction of ribs and the relatively small body size. However, salamanders are the only available postural model for early tetrapods among extant taxa [207,208] and were therefore considered herein despite these postcranial differences. 2) All species represent a mosaic of plesiomorphic and apomorphic features [groundplan; [2]]. For example, extant agnathans resemble early craniates such as the conodonts in their myomeric organization of their axial muscles or the possession of a notochord as the main axial skeleton [69]. However, they are highly specialized relicts of a multifaceted group of jawless craniates that possessed for example dermal armor to a varying extent [i.e., ostracoderms, [69]]. Therefore, inference of plesiomorphic axial muscle characteristics for craniates is potentially confounded by derived character states in extant hagfish and lampreys. 3) The depth to which we know intra-taxon variation and the confidence with which we can infer the set of character states in the common ancestor of the respective groups varies greatly. Groups such as actinopterygians or mammals have been investigated intensively. Therefore, their interspecific variability and the adaptive value of the various muscular arrangements are fairly well-understood. In such groups, we can start sorting out character states that represent phylogenetic history from those that are more likely the immediate result of adaptation. Only very few species have been studied so far in other groups such as salamanders or lizards and the ground-plan set of character states may not be unequivocal yet. Some caution is required when species from such groups are used to infer character states in ancestors as the full extend of within-group muscular variation has not been established yet. However, this considered, inclusion of the currently known evidence in hypotheses as stated herein provides a clear framework for future hypothesis driven research with options for falsification.

## Competing interests

The authors declare that they have no competing interests.

## Authors' contributions and information

Part of this manuscript is based on the author's Habilitation-Thesis. Thanks to the organizers of the 103. Annual Meeting of the German Zoological Society as well as to M. Nickel and C.S. Wirkner for their invitation to the symposium and the opportunity to publish this review.
